# Phenotypes of Food Allergies in Patients with Atopic Dermatitis Aged Under 24 Months: A Multicenter Study

**DOI:** 10.3390/diagnostics15202656

**Published:** 2025-10-21

**Authors:** Mujde Tuba Cogurlu, Metin Aydogan, Ozlem Cavkaytar, Pinar Uysal, Hazal Cansu Culpan, Nalan Yakici, Zeynep Hizli Demirkale, Erdem Topal, Hasan Yuksel, Sezin Aydemir, Nursen Cigerci Gunaydin, Cigdem Aydogmus, Sukru Cekic, Emre Akkelle, Tuba Tuncel, Isil Eser Simsek, Mustafa Arga, Zeynep Ülker Altinel, Fatih Kaplan, Ayca Kiykim, Ayse Suleyman, Nermin Guler, Esra Yucel, Haluk Cokugras, Nihat Sapan, Hikmet Tekin Nacaroglu, Adem Yasar, Yakup Yesil, Gonca Hancioglu, Recep Sancak, Mehmet Sarper Erdogan, Oner Ozdemir, Cevdet Ozdemir, Fazil Orhan

**Affiliations:** 1 Department of Pediatric Allergy and Immunology, Sakarya Training and Research Hospital, 54100 Sakarya, Türkiye; 2 Department of Pediatric Allergy and Immunology, Faculty of Medicine, Kocaeli University, 41000 Kocaeli, Türkiye; metin.aydogan@kocaeli.edu.tr (M.A.);; 3 Department of Pediatric Allergy and Immunology, Faculty of Medicine, Istanbul Medeniyet University, 34000 Istanbul, Türkiye; 4 Department of Pediatric Allergy and Immunology, Faculty of Medicine, Aydin Adnan Menderes University, 09000 Aydin, Türkiye; 5 Department of Public Health, Cerrahpasa Faculty of Medicine, Istanbul University-Cerrahpasa, 34000 Istanbul, Türkiye; 6 Department of Pediatric Allergy and Immunology, Faculty of Medicine, Karadeniz Teknik University, 61000 Trabzon, Türkiye; 7 Division of Pediatric Allergy and Immunology, Faculty of Medicine, Istanbul University, 34000 Istanbul, Türkiye; 8 Department of Pediatric Allergy and Immunology, Faculty of Medicine, Inonu University, 44000 Malatya, Türkiye; 9 Department of Pediatric Allergy and Immunology, Faculty of Medicine, Celal Bayar University, 45000 Manisa, Türkiye; 10 Department of Pediatric Allergy and Immunology, Faculty of Medicine, Istanbul University-Cerrahpasa, 34000 Istanbul, Türkiye; 11 Department of Pediatric Allergy and Immunology, Faculty of Medicine, Namık Kemal University, 59000 Tekirdag, Türkiye; 12 Department of Pediatric Allergy and Immunology, Kanuni Sultan Suleyman Training and Research Hospital, 34000 Istanbul, Türkiye; 13 Department of Pediatric Allergy and Immunology, Faculty of Medicine, Uludag University, 16000 Bursa, Türkiye; 14 Department of Pediatric Allergy and Immunology, Medical Park Hospital, 34000 Istanbul, Türkiye; 15 Department of Pediatric Allergy and Immunology, Faculty of Medicine, İzmir Katip Celebi University, 34000 Izmir, Türkiye; 16 Department of Pediatric Allergy and Immunology, Faculty of Medicine, Medipol University, 34000 Istanbul, Türkiye; 17 Department of Pediatric Allergy and Immunology, Faculty of Medicine, Ondokuz Mayıs University, 55000 Samsun, Türkiye; 18 Department of Pediatric Allergy and Immunology, Faculty of Medicine, Sakarya University, 34000 Sakarya, Türkiye

**Keywords:** atopic dermatitis, food allergy, oral food challenge, non-IgE-mediated

## Abstract

**Background:** Atopic dermatitis (AD) and food allergy (FA) are common allergic diseases in early childhood. AD may be concomitant with FA, particularly in young children. Although studies report the prevalence of FA in children with AD, there is insufficient data regarding different phenotypes of FA. **Objective:** The aim of our research was to determine the prevalence and clinical predictors of different phenotypes of concomitant FA in children with AD. **Methods:** This cross-sectional multicenter study included patients younger than 24 months old diagnosed with AD, recruited from 14 pediatric allergy centers. Patients were categorized into two groups using skin testing, allergen-specific IgE, and ultimately food challenge testing (FCT): those with FA and those without. Individuals with FA were classified into three distinct phenotypes: IgE-mediated, non-IgE-mediated, and concurrent IgE- and non-IgE-mediated. **Results:** The data of 530 children [59% male, median-age 7 months (IQR: 5–11)] were analyzed. IgE-mediated FA was found in 28.1% of participants, whereas 22.4% (*n* = 119/530) exhibited non-IgE-mediated FA. Concurrent IgE- and non-IgE-mediated FA was reported in 12.1% (*n* = 64/530) of patients. Cow’s milk (69.6%) and egg-white (68.9%) were identified as the most prevalent allergens. Cow’s milk was primarily responsible for non-IgE-mediated and egg-white for IgE-mediated FA. The most significant predictors of FA were severe AD and the presence of blood in stool with odds ratios of 8.25 (95% Cl: 3.04–22.39) and 10.04 (95% CI: 2.03–49.59), respectively (*p* < 0.01) (*p* < 0.005). **Conclusions:** The study’s findings indicate that children with early-onset and mild-to-moderate AD deserve to be comprehensively assessed for FA symptoms. The most significant indicators of concomitant FA in AD patients were the presence of blood in stool and severe AD. It is important to consider that those who exhibit IgE-mediated FA may also have concurrent non-IgE-mediated FA. We underline that it is important to consider that children with AD who exhibit IgE-mediated FA may also have concurrent non-IgE-mediated FA. Addressing these symptoms may assist healthcare practitioners in clinical practice to improve the quality of care for AD patients having FA.

## 1. Introduction

Atopic dermatitis (AD) and food allergy (FA) are common allergic diseases in early childhood, and the prevalences of both have increased in the last decade [1]. There is an increased risk of FA in children with AD, particularly in those less than 24 months [2,3]. FA can develop through various mechanisms in this population, such as IgE- or non-IgE-mediated ones, and manifest different clinical symptoms from each other [4]. The timely and precise identification of FA phenotypes is essential for managing disease progression. Conversely, the exclusion of FA is crucial to prevent unnecessary elimination diets and adverse clinical outcomes [5]. Data regarding the prevalence of concomitant IgE-mediated FA in AD patients are available due to the objective nature of symptoms and the utility of molecular allergology in the diagnostic process [6]. There remains an insufficient amount of knowledge regarding various phenotypes, especially non-IgE-mediated and concurrent IgE- and non-IgE-mediated FA [7].

The main objective of our study was to determine the prevalence of different phenotypes of concomitant FA in AD patients under 24 months. We also aimed to identify clinical predictors of FA.

## 2. Methods

This cross-sectional multicenter study included patients younger than 24 months old diagnosed with AD, recruited from 14 pediatric allergy centers in tertiary hospitals in Turkey between March 2019 and February 2020. Ethical approval was obtained from Kocaeli University Medical Faculty Clinical Trials Ethics Committee (project no: 2019/11, decision no: KU GOKAEK 2019/01.17, date of approval: 1 September 2019). The study was conducted in compliance with the Declaration of Helsinki of 1975 (revised in 2013), and parents provided written informed consent before recruitment to the study.

### 2.1. Data Acquisition and Patient Follow-Up

Individuals under 24 months of age diagnosed with AD by clinicians based on the Hanifin-Rajka criteria were included in the study [8]. Exclusion criteria included the presence of a physician-diagnosed primary immune deficiency or a significant systemic disease affecting other organ systems. The initial data comprised baseline demographic characteristics and a comprehensive clinical history of AD. Furthermore, parents were inquired about the presence of particular dietary proteins that might be responsible for the symptoms related to FA in the mother’s diet (for breast-fed children) or in the patient’s diet [9].

**Figure 1:**
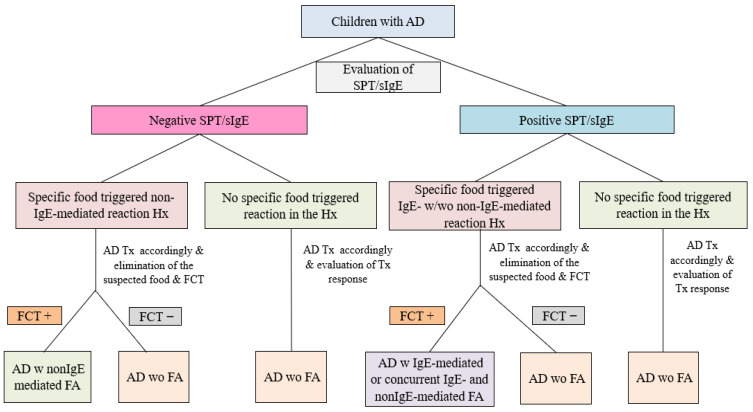
Flow chart of the study. AD: Atopic Dermatitis, FA: Food Allergy, sIgE: specific immunoglobulin E, SPT: Skin Prick Test, Hx: History, Tx: Treatment, w: With, wo: Without, FCT: Food Challenge Test.

The severity of AD was assessed using the Scoring Atopic Dermatitis (SCORAD) index and was classified as mild (0–25 points), moderate (>25–50 points), or severe (>50 points) [10]. Skin prick tests (SPT)/serum specific immunoglobulin E (sIgE) were conducted on all patients for cow’s milk (CM), egg-white, peanut, tree nuts (hazelnut and walnut), wheat, beef, sesame, lentil, soy, and fish [11]. All patients underwent consistent treatment for AD with moisturizers and topical corticosteroids, followed by a re-evaluation of treatment responses after a two-week period [12]. Open oral food challenge tests (FCT) were performed a minimum of 15 days following the resolution of AD lesions and other FA-related symptoms [13]. FCTs were conducted on all patients with a history of specific food-triggered IgE-mediated reactions under physician supervision in a hospital environment. Patients with histories of non-IgE-mediated food-induced symptoms, lacking food sensitization, were instructed to consume the index food at home following the elimination period. They were then re-evaluated for the occurrence of non-IgE-mediated gastrointestinal symptoms during the 2–4 weeks following OFC [14]. The food identified as responsible, in accordance with OFC, was removed from the patients’ diet (Figure 1).

### 2.2. Classification of FA Phenotypes

- AD concomitant IgE-mediated FA

Patients demonstrating a positive SPT/sIgE, in conjunction with a positive FCT indicated by the emergence of at least one IgE-mediated symptom (urticaria, angioedema, vomiting within 2 h, respiratory symptoms, or anaphylaxis) or an exacerbation of AD indicated by a minimum 10-point increase in the SCORAD index, were categorized as “AD concomitant IgE-mediated FA.”

- AD concomitant non-IgE-mediated FA

Patients exhibiting symptoms of non-IgE-mediated FA (food protein-induced allergic proctocolitis), such as blood in stool, recurrent vomiting, loose stools, mucus in stool, painful flatulence, and colic, while being SPT/sIgE negative, were subjected to a diet including the suspected food for a duration of two weeks. Patients experiencing the same reactions following FCT with the suspected food after a two- to four-week elimination period were categorized as “AD concomitant non-IgE-mediated FA.”

- AD concomitant IgE- and non-IgE-mediated FA

Individuals who responded with both an IgE- and a non-IgE-mediated FA to a particular food protein or to various food proteins are classified as “AD concomitant IgE and non-IgE-mediated FA.”

- AD without FA

Patients who exhibit negative SPT/sIgE, or those with positive SPT/sIgE without experiencing any symptoms or exacerbation of AD during OFC after elimination for 7 days, or who do not indicate non-IgE-mediated symptoms and respond positively to standard AD treatment during follow-up for 4 weeks, are classified as “AD without FA.”

## 3. Statistical Analysis

SPSS v21.0 (SPSS Inc., Chicago, IL, USA) and Microsoft Office Excel (Microsoft Corporation, Redmond, WA, USA) software were used for statistical analysis. Continuous variables were presented as median and interquartile range (IQR), and categorical variables as frequency and percentage. The Chi-square test or Fisher Exact test, as appropriate, was used to analyze categorical variables. Comparison of continuous variables between two groups was performed using the Mann–Whitney U test and with the Kruskal–Wallis test for more than two groups. Post hoc tests were used for pairwise comparisons. A *p*-value < 0.05 was regarded as statistically significant. Multivariate logistic regression analysis was performed to explain the co-existence of FA with AD. Results were presented as Odds Ratio (OR) and 95% Confidence Interval (95% CI). The Receiver Operating Characteristic (ROC) curve method was used, and area under the curve (AUC) values were given.

## 4. Results

### 4.1. Demographic and Clinical Characteristics of AD Patients, with or Without FA

**Figure 2:**
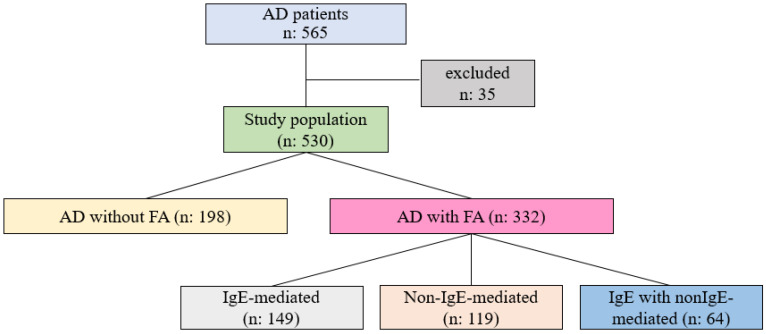
Flow diagram of the participants.

Of the 565 participants, 35 were excluded from the study based on factors such as insufficient follow-up (*n* = 11) and failure to undergo (declined consent) FCT (*n* = 24) (Figure 2). The study was completed with 530 individuals diagnosed with AD with a median age of 7 months (IQR: 5–11), of whom 59.4% (*n* = 315) were male. The subjects classified as “AD concomitant FA” (62.6%, *n* = 332) had a median age upon diagnosis of 7 months (IQR: 5–10.8), with 59% being male. In a comparison of patients’ median ages (2 months, IQR: 1–3.5) at the onset of AD symptoms, individuals with AD concomitant FA were significantly younger than those without FA (3 months, IQR: 2–6) (*p* < 0.001). Patients with FA exhibited more severe AD, with a SCORAD median of 21.9 (IQR: 15.5–31.1), compared to individuals without FA, who also had a SCORAD median of 31.3 (IQR: 20.6–45) (*p* < 0.001) (Table 1).

### 4.2. Clinical Characteristics of FA Phenotypes

Three hundred eighty-six patients underwent 529 FCTs, and FA was confirmed in 332 (62.6%) patients. The analysis determined that 4 months is the most discriminative age for distinctive variables related to various types of FA in the study population. AD concomitant IgE-mediated FA was identified in 28.1% (*n* = 149/530) of participants. FCT results indicated that isolated urticaria occurred in 59 patients, vomiting in 6 patients, respiratory symptoms in 4 patients, and anaphylaxis in 5 patients. Of the participants, 22.4% (*n* = 119/530) were identified as exhibiting non-IgE-mediated FA. During the late period, blood in stool was noted in 50 patients, intermittent vomiting in 30 patients, and loose stool in 24 patients. Concurrent IgE- and non-IgE-mediated FA was observed in 12.1% (*n* = 64/530) of the patients with AD (Table 2).

### 4.3. The Distribution of Triggering Foods for Different Phenotypes of FA

**Figure 3:**
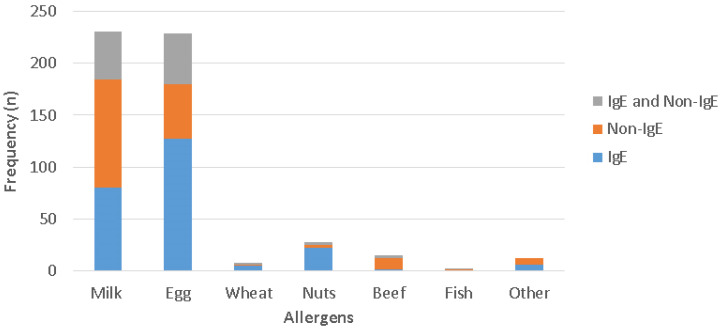
The different triggers of FA according to various FA phenotypes in AD patients.

Despite a negative FCT, positive sIgE/SPT results were observed in 27.2% (*n* = 54/198) of AD patients without FA. Approximately 34.6% (*n* = 80/231) of patients with CM allergy (CMA) exhibited IgE-mediated responses, while 45.0% (*n* = 104/231) had only non-IgE-mediated reactions, and 20.3% (*n* = 47/231) presented with concurrent IgE- and non-IgE-mediated FA. Among patients with egg-white allergy, 55.5% (*n* = 127/229) exhibited IgE-mediated, 23.1% (*n* = 53/229) demonstrated non-IgE-mediated, and 21.4% (*n* = 49/229) presented with both IgE- and non-IgE-mediated reactions. The majority of patients with peanut and tree nut allergy (78.6%, *n* = 22/28) had IgE-mediated FA (Figure 3).

### 4.4. Clinic Predictors of FA Phenotypes

**Figure 4:**
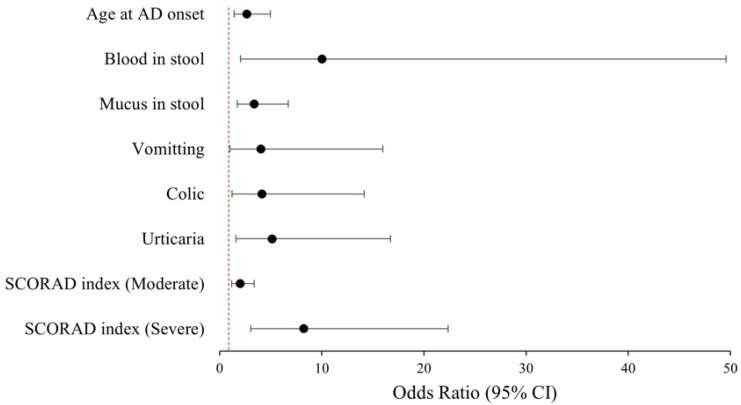
Forest plot of factors predicting concurrent food allergy in patients with atopic dermatitis (The red dashed line indicates OR = 1).

The analysis revealed that the probability of concomitant FA with AD was significantly increased by factors including moderate-to-severe AD, the presence of blood in stool, mucus in stool, symptoms of urticaria, vomiting, colic, and the emergence of AD at or before 4 months of age. Multivariate logistic regression analysis indicated that the most significant predictors of the coexistence of FA in AD patients were the presence of blood in stool and severe AD with OR of 10.04 (95% CI: 2.03–49.59) and 8.25 (95% CI 3.04–22.39), respectively (Table 3, Figure 4).

## 5. Discussion

Our research conducted on a population under 24 months of age found that the prevalence of FA is 62.7% among children with AD. Multiple studies demonstrate that children diagnosed with moderate-to-severe AD at a younger age are more likely to have concomitant FA [15,16,17].

Our study revealed that 87.9% of patients identified with FA exhibited the onset of AD before 4 months of age. Mild AD was observed in 58% of patients without FA, whereas 65.7% of patients with FA exhibited moderate-to-severe AD. This figure may primarily result from the study’s setting in tertiary allergy centers and the observation that over half of the children with FA exhibited moderate-to-severe AD. The majority of patients with mild AD can be treated in primary care and are not referred to tertiary allergy centers.

The primary objective of this research is to highlight the different FA phenotypes in children with AD. The prevalence of IgE-mediated FA varies across studies, ranging from 17.8% to 40% when diagnoses were confirmed by FCTs. This rate increases from 33% to 81% in patients with moderate-to-severe AD [18,19,20]. The present study found that the frequency of solely IgE-mediated FA was 28.1%. Recognition of IgE-mediated FA is more straightforward due to the presence of objective symptoms and the availability of standardized diagnostic tests (SPT, sIgE, OFC, etc.) [21]. Conversely, diagnostic tests for non-IgE-mediated FA are infrequent and lack standardization [22]. Therefore, the data concerning the frequency of non-IgE-mediated FA in patients with AD remains inadequate [23,24]. One of the most important results of our study is that the rate of non-IgE-mediated FA was found to be 22.4%, closely approximating the ratio of IgE-mediated FA. These results indicate that in young children with AD, the existence of non-IgE-mediated FA symptoms should be disclosed with a detailed investigation.

The literature lacks definitive data regarding the concurrent IgE- and non-IgE-mediated FA in children with AD [7]. This coexistence was observed in 12.0% of individuals. We would like to emphasize that, in addition to immediate reactions, symptoms such as blood and/or mucus in the stool, vomiting, and colic should be considered for the risk of non-IgE-mediated FA in AD patients.

Although the prevalence of food sensitization among children with AD has been reported to range from 47% to 66% in the literature, it is known that the frequency of allergy is lower [25,26,27]. Our study found that 27% of patients without FA exhibited sIgE/SPT positivity, despite negative FCT results. The FCT test, which is the gold standard in the diagnosis of FA, is strongly recommended to diagnose true FA and to prevent unnecessary food elimination and adverse clinical outcomes, especially in AD patients with food sensitivity [22]. FCT decisions may not always be straightforward for individuals and healthcare professionals. Therefore, it is essential to identify who is at high risk for FA [28]. The risk of FA is known to rise in the presence of early-onset and moderate-to-severe AD [29,30]. We established that the presence of blood in stool was the most significant risk factor for FA, alongside early-onset and moderate-to-severe AD.

The main strengths of the study are its multicenter prospective design, extensive sample size, and the confirmation of FA by pediatric allergy specialists with FCT. The simultaneous evaluation of immediate and late-onset symptoms is another strength. Open FCTs may be regarded as a drawback; however, the patients in our study sample were under 2 years of age, allowing for objective assessment of FCTs in this demographic. The fact that our study population consisted of AD patients under 24 months of age could be considered a limitation. But we aimed to contribute to the literature in this area due to the higher prevalence of the association between AD and FA in this age group.

## 6. Conclusions

Our research offers important insights about the concomitant FA phenotypes in children with AD in early childhood. Children with early-onset and mild-to-moderate AD should be thoroughly investigated for FA symptoms. Diagnosing true FA is crucial in order to avoid unnecessary food elimination and negative clinical consequences, particularly in AD patients who have food sensitivity. The findings of the study suggest that the primary indicators of concomitant FA in AD patients were the presence of blood in stool and severe AD. We underline that it is important to consider that children with AD who exhibit IgE-mediated FA may also have concurrent non-IgE-mediated FA. Addressing these symptoms may assist healthcare practitioners in clinical practice to improve the quality of care for AD patients having FA.

## Figures and Tables

**Table 1:** Demographic and clinical characteristics of atopic dermatitis cases (*n* = 530) with and without food allergy.

	AD Without FA (*n* = 198)	AD with FA (*n* = 332)	*p* Value
Age (months) ^†^	7.5 (5.5–12.5)	7 (5–10.8)	0.008 *
Age at AD onset (months) ^†^	3 (2–6)	2 (1–3.5)	<0.001 *
Sex (male), *n* (%)	119 (60.1)	196 (59)	0.809 **
SCORAD index ^†^	21.9 (15.5–31.1)	31.3 (20.6–45)	<0.001 *
AD severity (*n*, %)			<0.001 **
Mild	116 (58.6)	114 (34.3)	
Moderate	75 (37.9)	161 (48.5)	
Severe	7 (3.5)	57 (17.2)	
Parental history of AD, *n* (%)	24 (12.1)	54 (16.3)	0.193 **

AD: Atopic dermatitis, FA: Food Allergy, SCORAD: Scoring atopic dermatitis index. ^†^ Median (interquartile range). * Mann–Whitney U test. ** Chi-square test.

**Table 2:** Clinical characteristics of atopic dermatitis cases (*n*:530) without food allergy and concomitant various food allergy phenotypes.

	AD Without FA (*n* = 198)	AD Concomitant IgE-Mediated (*n* = 149)	AD Concomitant Non-IgE-Mediated (*n* = 119)	AD Concomitant IgE with Non-IgE-Mediated (*n* = 64)	*p* Value
Age (months) ^†^	7.5 (5.5–12.5)	7.5 (5.5–11.3)	5.5 (4.5–8.5)	6.5 (4.5–10.8)	<0.001 ^‡^
Age at onset of AD (months) ^†^	3 (2–6)	2 (1–4)	2 (1–3)	2 (1–3)	<0.001 ^§^
Age at onset of AD ≤ 4 months, *n* (%)	131 (66.2)	126 (84.6)	105 (88.2)	61 (95.3)	<0.001 ^¶^
Sex (male), *n* (%)	119 (60.1)	94 (63.1)	61 (51.3)	41 (64.1)	0.193 ^¶^
SCORAD index ^†^	21.8 (15.5–31.1)	32.4 (21.2–48.5)	26.9 (18.6–35.2)	35.6 (28–49.8)	<0.001 *
AD severity, *n* (%)					<0.001 ^¶^
Mild	116 (58.6)	52 (34.9)	50 (42.1)	12 (18.8)	
Moderate	75 (37.9)	66 (44.3)	58 (48.7)	37 (57.8)	
Severe	7 (3.5)	31 (20.8)	11 (9.2)	15 (23.4)	
Feeding types at diagnosis, *n* (%)					
Breastfeeding	140 (71.8)	132 (90.4)	106 (91.4)	59 (95.2)	<0.001 ^¶^
Formula feeding	36 (18.6)	22 (15.1)	37 (31.9)	17 (27.4)	0.004 ^¶^
Complementary feeding	59 (30.3)	35 (24.1)	15 (12.9)	5 (8.1)	<0.001 ^¶^
Other FA related symptoms in the history, *n* (%)					
Blood in stool	2 (1)	2 (1.3)	37 (31.1)	17 (26.6)	<0.001 ^¶^
Mucus in stool	21 (10.6)	26 (17.4)	76 (63.9)	30 (46.9)	<0.001 ^¶^
Vomiting	3 (1.5)	12 (8.1)	24 (20.2)	12 (18.8)	<0.001 ^¶^
Colic	4 (2)	13 (8.7)	37 (31.1)	14 (21.9)	<0.001 ^¶^
Urticaria	5 (2.5)	26 (17.4)	2 (1.7)	9 (14.1)	<0.001 ^¶^
Angioedema	0	3 (2)	0	2 (3.1)	0.019 **
Anaphylaxis	0	5 (3.4)	0	0	0.05 **

AD: Atopic Dermatitis, FA: Food Allergy, SCORAD: Scoring atopic dermatitis index. ^†^ Median (interquartile range). ^‡^ Kruskal–Wallis test. Non-IgE-mediated group is different from AD without FA and IgE-mediated groups. ^§^ Kruskal–Wallis test. AD without FA group is different from others. ^¶^ Chi-square test. * Kruskal–Wallis test. All groups are different from each other except IgE-mediated and IgE concurrent non-IgE-mediated-FA groups. ** Fisher Exact test.

**Table 3:** Multivariate logistic regression of factors predicting concurrent food allergy in patients with atopic dermatitis.

	OR	95% CI	*p* Value
Age at AD onset (≤4 months)	2.67	1.43–4.98	0.002
Blood in stool	10.04	2.03–49.59	0.005
Mucus in stool	3.39	1.72–6.71	<0.001
Vomitting	4.02	1.01–15.98	0.048
Colic	4.14	1.21–14.15	0.024
Urticaria	5.14	1.58–16.72	0.007
SCORAD index (referring mild AD)			
Moderate	2.01	1.19–3.39	0.009
Severe	8.25	3.04–22.39	<0.001

AD: Atopic Dermatitis, OR: Odds Ratio, SCORAD: Scoring atopic dermatitis index.

## Data Availability

The original contributions presented in this study are included in the article. Further inquiries can be directed to the corresponding author.

## Abbreviations

AD	Atopic Dermatitis
AUC	Area under the curve
CM	Cow’s milk
CMA	Cow’s milk allergy
CI	Confidence Interval
FA	Food Allergy
FCT	Food challenge test
IQR	Interquartile range
OR	Odds Ratio
ROC	The Receiver Operating Characteristic
SCORAD	Scoring of Atopic Dermatitis
SI	SCORAD Index
sIgE	Specific Immunoglobulin E
SPT	Skin prick test
